# Different behaviour of C-banded peri-centromeric heterochromatin between sex chromosomes and autosomes in Polyphagan beetles

**DOI:** 10.3897/CompCytogen.v13i2.34746

**Published:** 2019-07-03

**Authors:** Anne-Marie Dutrillaux, Bernard Dutrillaux

**Affiliations:** 1 UMR7205 MNHN CNRS UMPC EPHE Institut de Systématique, Evolution, Biodiversité. Muséum National d’histoire Naturelle, Sorbonne Universités, 57, rue Cuvier, CP39, UMR7205 Paris, France Sorbonne Universités Paris France

**Keywords:** Coleoptera, Polyphaga, karyotypes, heterochromatin, variation, sex chromosomes

## Abstract

Heterochromatin variation was studied after C-banding of male karyotypes with a XY sex formula from 224 species belonging to most of the main families of Coleoptera. The karyotypes were classified in relation with the ratio heterochromatin/euchromatin total amounts and the amounts of heterochromatin on autosomes and gonosomes were compared. The C-banded karyotypes of 19 species, representing characteristic profiles are presented. This analysis shows that there is a strong tendency for the homogenization of the size of the peri-centromeric C-banded heterochromatin on autosomes. The amount of heterochromatin on the X roughly follows the variations of autosomes. At contrast, the C-banded heterochromatin of the Y, most frequently absent or very small and rarely amplified, looks quite independent from that of other chromosomes. We conclude that the Xs and autosomes, but not the Y, possibly share some, but not all mechanisms of heterochromatin amplification/reduction. The theoretical models of heterochromatin expansion are discussed in the light of these data.

## Introduction

There is a consensus to consider that the ancestral karyotype of Polyphagan beetles was composed of 20 chromosomes, a number observed in living specimens from most families (Smith and Virkki 1978). The sex chromosomes, XX and XY in females and males, respectively, are usually among the smallest chromosomes. Occasional size increases have recurrently been reported, but the origin of these increases has never been systematically investigated. Considering the large compilation of Smith and Virkki (1978), the rate of enlarged sex chromosomes, generally referred to as neo sex chromosomes, was estimated at 8.3% of species (Dutrillaux and Dutrillaux 2009), but the formal distinction between the sex chromosomes derived from a translocation with autosomal material and those having amplified their heterochromatin content could not be made in most ancient publications. On the whole, the use of chromosome banding remains limited in beetles. Their euchromatin does not contain large fragments of repetitive DNA sequences, such as LINES and SINES in mammals, which probably originate G and R bands, after appropriate treatments (Bickmore and Craig 1997). This explains the high compaction of the genome in beetles, in which the gene density is many-fold that of mammals (Dutrillaux 2016). Thus, beside techniques of molecular cytogenetics using satDNA probes, which were applied on some species (Petitpierre 1980, Pons et al. 2002, 2004), only C-banding can be regularly achieved for detecting heterochromatin, which harbors highly repeated DNA. However, it remains poorly efficient in some families, such as Cerambycidae, in which centromere regions often remain poorly or not C-banded (Dutrillaux and Dutrillaux 2018). Silver staining, generally used for the detection of nucleolar proteins at contact with the NOR (Nucleolus Organizer Region), frequently stains a portion of heterochromatin in beetles. DNA replication studies, which allow a differentiation between late replicating heterochromatin and early replicating euchromatin are difficult to apply in the absence of cell culture and remain exceptionally used (see below). In spite of these difficulties, we tried to find some rules governing heterochromatin variation in beetles, especially that of the sex chromosomes, in relation with that of autosomes. For this purpose, we analyzed male specimens of 344 species of Polyphagan beetles, for which C-banding was systematically applied. It will be shown that, as regard their heterochromatin content, the X and the Y have a very different behavior.

## Material and methods

### Insects

We collected most of the specimens from the 344 studied species in France, Greece and West Indies. Some specimens were also obtained from amateur breedings, Besançon insectarium, or kindly provided by colleagues and friends. The species studied here were distributed into 21 families, but most belonged to Cerambycidae (67 species), Chrysomelidae (40 species), Curculionidae (18 species), Lucanidae (11 species), Scarabaeidae (136 species) and Tenebrionidae (28 species). We established the karyotype of the 344 species, among which we selected the19 following species, as examples of the various situations observed:

*Adaliabipunctata* Linneaeus, 1758 (Coccinelidae, Coccinelinae) (France);

*Amphimallonsolstitiale* Linnaeus, 1758 (Scarabaeida, Melolonthinae) (France);

*Asidajurinei* Solier, 1836 (Tenebrionidae, Pimeliinae) (France);

*Criocerisasparagi* Linnaeus, 1758 (Chrysomelidae, Criocerinae) France;

*Cyclocephalapicipes* Olivier, 1789 (Scarabaeidae, Dynastinae) (French Guyana);

*Disonychalatifrons* Schaeffer, 1919 (Chrysomelidae, Alticinae) (Canada, Quebec); *Dorcadion* (*Cribridorcadion*) *etruscum* Rossi, 1790 (Cerambycidae, Lamiinae) (Italy);

*Lamprimaadolphinae* Gestro, 1875 (Lucanidae) (New Guinea);

*Leucothyreusnolleti* Paulian, 1947 (Scarabaeidae, Rutelinae) (Martinique);

*Liliocerislili* Scopoli, 1763 (Chrysomelidae, Criocerinae) (France);

*Lucanuscervus* Linneaeus, 1753 (Lucanidae) (France);

*Macraspistristis* Castelnau, 1840 (Scarabaeidae, Rutelinae) (Guadeloupe);

*Melolonthamelolontha* Linnaeus, 1758 (Scarabaeidae, Melolonthinae) (France);

*Melolonthahippocastani* Fabricius, 1801 (Scarabaeidae, Melolonthinae) (France);

*Morimusfunereus* Mulsant, 1862 (Cerambycidae; Lamiinae) (Greece);

*Propomacrusdavidi* Deyrolle, 1874 (Scarabaeidae, Euchyrinae) (China);

*Scarabaeusvariolosus* Fabricius, 1787 (Scarabaeidae, Scarabaeinae) (Greece);

*Strategussyphax* Fabricius, 1775 (Scarabaeidae, Dynastinae) (Guadeloupe);

*Ulomaretusa* Fabricius, 1801 (Tenebrionidae, Tenebrioninae) (Guadeloupe).

### Cytogenetic methods

After anaesthesia by ethyl acetate, testicular follicles were dropped into an aqueous solution of 0.88 M KCl where they remained for 15 min at room temperature. They were transferred into a micro-centrifuge tube (VWR International SAS, code 211-0033, Strasbourg, France) containing 0.5 ml of 0.55 M KCl (hypotonic) solution, where they were squashed and suspended using a piston pellet (VWR, code 045420) adjusted to the internal diameter of the tube. The volume of 0.55 M KCl was completed to 1.5 ml. After 10 min, they were centrifuged during 5 min at 800 g. The supernatant was replaced by Carnoy I fixative, in which the cells were suspended and left for at least 30 min. After one change of fixative, the cells were spread on wet and cold slides or conserved for a few days before use. Slides were stained by Giemsa, photographed and C-banded according to Angus (1982). Many studies were also performed on mid-gut cells, according to Angus (1988). In addition, a prolonged hypotonic shock was applied for pachytene stage obtaining (Dutrillaux et al. 2006). For DNA replication studies on *Criocerisasparagi*, BrdU (5-bromodeoxyuridine) was added to the 0.88 M KCl solution (final concentration 20 mg/l) for 4h before the hypotonic shock. Slides were stained by acridine orange (Dutrillaux et al. 1973) and observed in fluorescence. Staining by quinacrine mustard was performed according to Caspersson et al. (1970).

### Evaluation of heterochromatin amplification

**Figure 1.**
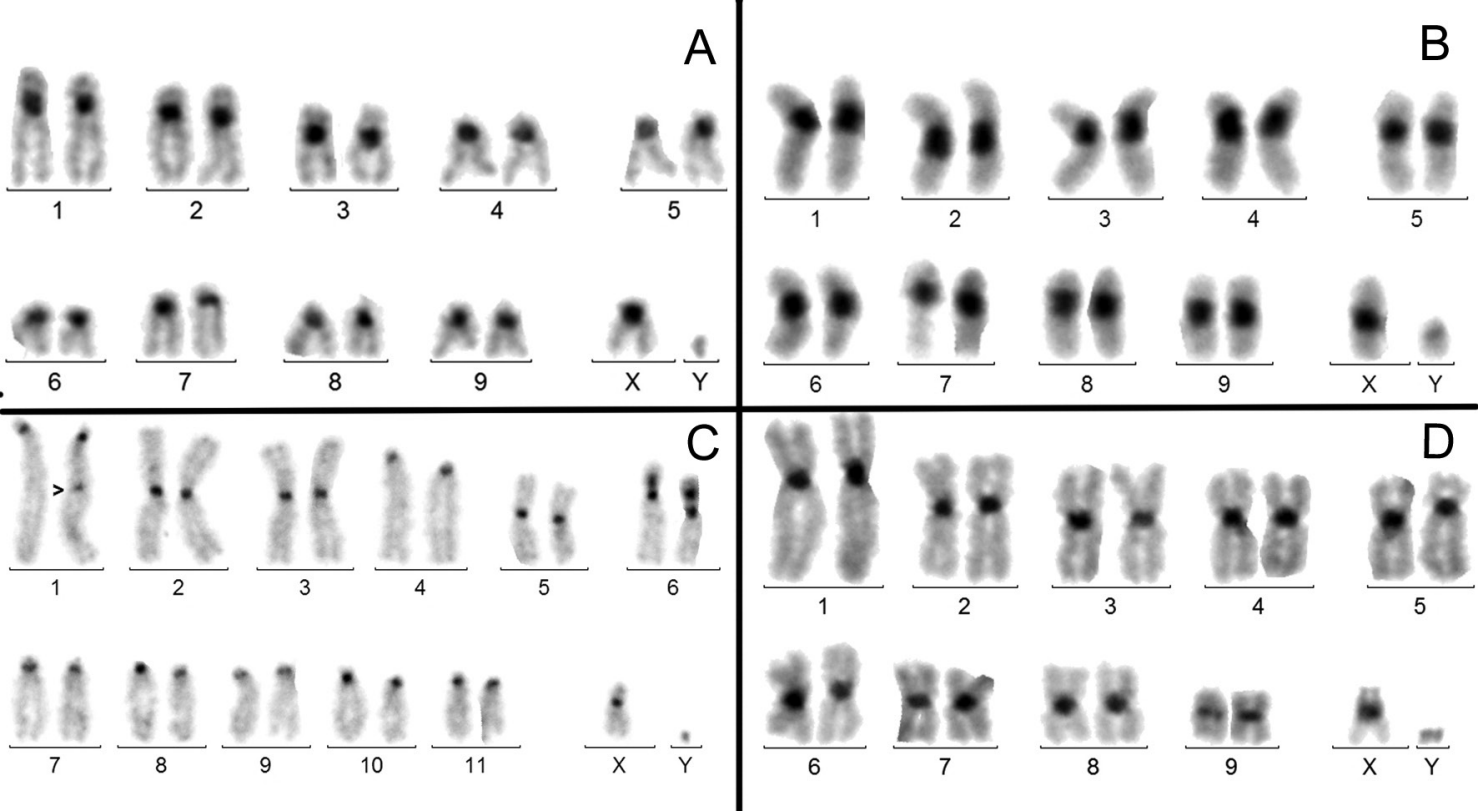
C-banded male karyotypes. **A***Adaliabipunctata***B***Asidajurinei***C***Morimusfunereus***D***Cyclocephalapicipes*. The autosomes and the X chromosomes have similar amounts of C-banded heterochromatin, but the Y chromosomes remain unstained.

**Figure 2.**
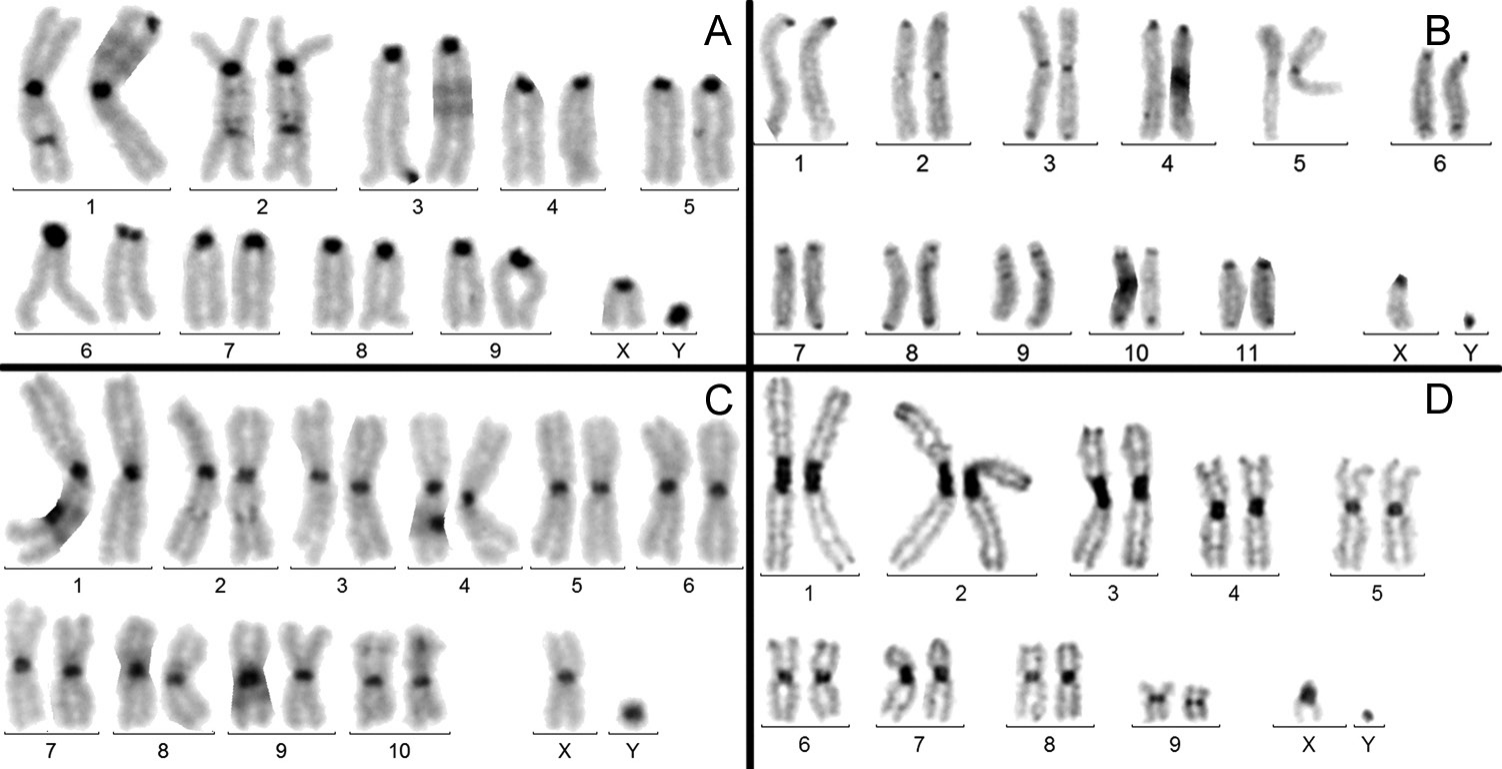
C-banded male karyotypes. **A***Amphimallonsolstitiale***B***Dorcadionetruscum***C***Liliocerislili***D***Strategussyphax*. In each karyotype, all centromere regions are similarly C-banded, but that of the Y is more variable.

**Figure 3.**
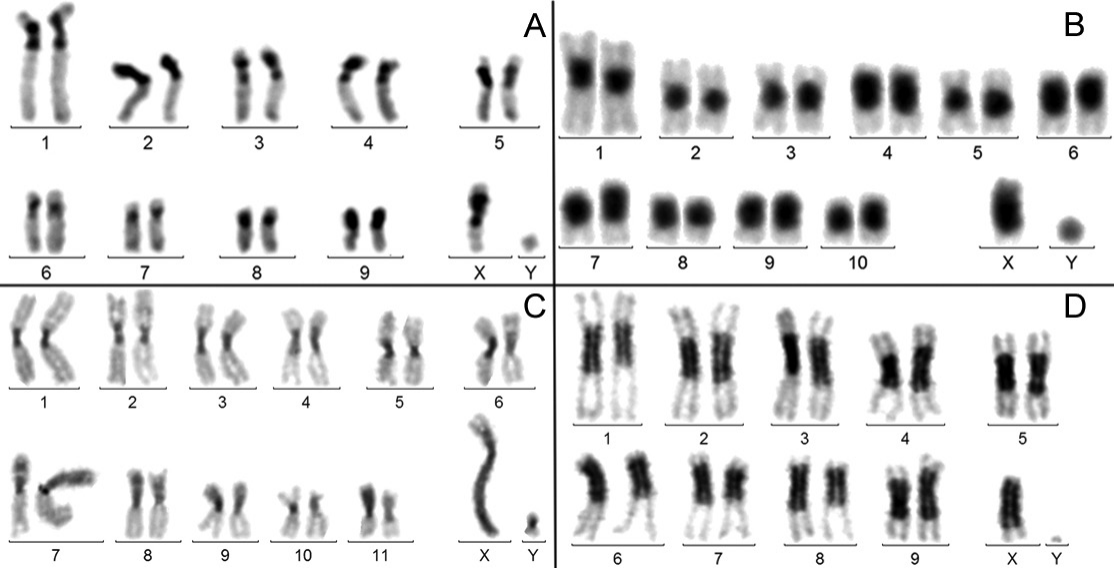
C-banded males karyotypes. **A***Ulomaretusa***B***Lucanuscervus***C***Disonychalatifrons***D***Melolonthahippocastani*. The level of heterochromatin amplification is often similar in the X and autosomes (**A, B, D**). The amplification may also be scattered, as in **C**, but it rarely involves the Y chromosome.

Not all heterochromatin is stainable by C-banding, but for technical reasons, only C-band positive heterochromatin will be considered. The usual intra- and inter-specific variation of heterochromatin makes it somewhat arbitrary to decipher its amplification. At the level of the whole karyotype, we have visually considered that heterochromatin is not amplified (NAH) when its amount represents less than 10% of the total chromosome length (Fig. 1A, C, D). It was considered as mildly amplified (MAH) when its total length was comprised between 10% and 25% that of chromosomes (Fig. 1B, 2D) and highly amplified (HAH) above 25% (Fig. 3A, B, D). Physical measurements were performed for ambiguous evaluations only. At the level of individual chromosomes, heterochromatin will be considered as amplified when its length is twice that of the average of other chromosomes of the karyotype (chromosome X, Fig. 3C).

## Results

For the above-mentioned species, this is the first report on C-banded karyotype, with the exception of *L.cervus*, *L.adolphinae*, *M.tristis*, *M.hippocastani*, *M.melolontha* and *S.syphax* (Giannoulis et al. 2011, Dutrillaux et al. 2007, 2012, Dutrillaux and Dutrillaux 2009). Some cytogenetic data, mainly chromosome counts, were also published for *A.solstitialis*, *A.jurinei*, *A.bipunctata*, *C.asparagi* and *L.lili* (John and Lewis 1960, Juan and Petitpierre 1991, Petitpierre 1980, Petitpierre et al. 1988, Virkki 1951).

Among the 344 male karyotypes studied, 25 (7.3%) without Y chromosome (X0 sex formula), 9 with a XYY formula (2.6%) and 35 (10.2%) with a gonosome-autosome translocation were excluded. Among the 275 remaining ones, the quality of the C-banding was considered to be sufficient for analysing both the size and the distribution of heterochromatin on chromosomes in 224 species. In this sample, a complete lack of C-banding on the Y chromosome was recorded in 134 instances (60%). At contrast, no C-banding was observed on the X chromosome in only 9 instances (4%). Among a large variety of profiles of heterochromatin distribution, some were particularly recurrent. They are listed below by order of decreasing occurrence.

**a) *Presence of clearly but not strongly amplified (NAH and MAH) C-banded heterochromatin on the centromere regions of all the chromosomes but the Y*.** It was observed in 86/224 instances (38.4%). Four examples are given in figure 1 in species from different families: *A.bipunctata* (Fig. 1A); *A.jurinei* (Fig. 1B); *M.funereus* (Fig. 1C) and *C.picipes* (Fig. 1D). In these species, the amount of centromeric heterochromatin varies from NHA , as in *M.funereus*, to MAH, as in *A.jurinei*, but is fairly similar, from chromosome to chromosome within each karyotype. Thus, there is a indisputable homogenization of the C-band size between autosomes and X. The lack or very small amount of C-banding on the Y shows that its heterochromatin dynamics is independent from that of both the X and the autosomes.

**b) *Presence of a clearly but not strongly amplified (NAH and MAH) C-banded heterochromatin on the centromere regions of all the chromosomes including the Y.*** It was observed in 60 instances (27%). Four examples are given in figure 2: *A.solstitiale*; *D.etruscum*; *L.lili* and *S.syphax*. Here again, there is some homogenization of the size of C-bands on both autosomes and X chromosome, but the size of the C-band on the Y is more independent: large in *A.solstitiale* (Fig. 2A) and very, small in *S.syphax* (Fig. 2D) karyotypes.

**c) *Presence of large heterochromatic fragments (MAH and HAH) on both the autosomes and the X.*** It was observed in 28 instances (12.5%). In this condition, there is not a systematic homogenization of the heterochromatin size on the autosomes, as in *U.retusa* (Fig. 3A) and the X may exhibit a very large heterochromatic fragment, as in *D.latifrons* (Fig. 3C). In many species, however, the amplification of heterochromatin is roughly similar on the X and autosomes, as in *L.cervus* and *M.hippocastani* (Fig. 3B, D). The C-banding of the Y is poor or absent, thus completely independent from that of both the X and autosomes.

**Figure 4.**
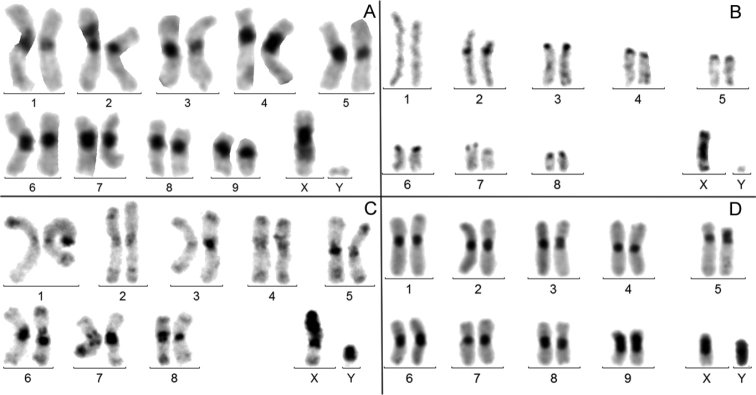
C-banded male karyotypes. **A***Leucothyreusnolleti***B***Propomacrusdavidi***C***Lamprimaadolphinae***D***Scarabaeusvariolosus*. Large heterochromatin amplification can involve the X alone (**A, B, C**) and more rarely the Y (**D**).

**d) *Presence of a large amplification of heterochromatin on the X chromosome but not on autosomes.*** It was observed in 25 species (11.2%). In these karyotypes, C-banded heterochromatin was either invisible on chromosome Y, as in *L.nolleti* and *P.davidi* (Fig. 4A, B), or present and even amplified, as in *L.adolphinae* (Fig. 4C).

**e) *Heterochromatin amplification on chromosome* Y.** It was noticed in 23 instances only (10.4%). Compared to both the X and autosomes, this amplification was almost always limited in size, some of the largest C-bands on the Y were observed in *S.variolosus* (Fig. 4D) and in species of Geotrupidae (not shown), as described by Wilson and Angus (2004). We recently found a very strong amplification of heterochromatin on both the X and Y in *Oxymiruscursor* (Cerambycidae, Lepturinae) but this species was not included in this study (Dutrillaux and Dutrillaux 2018).

### Intra-specific variation of heterochromatin

**Figure 5.**
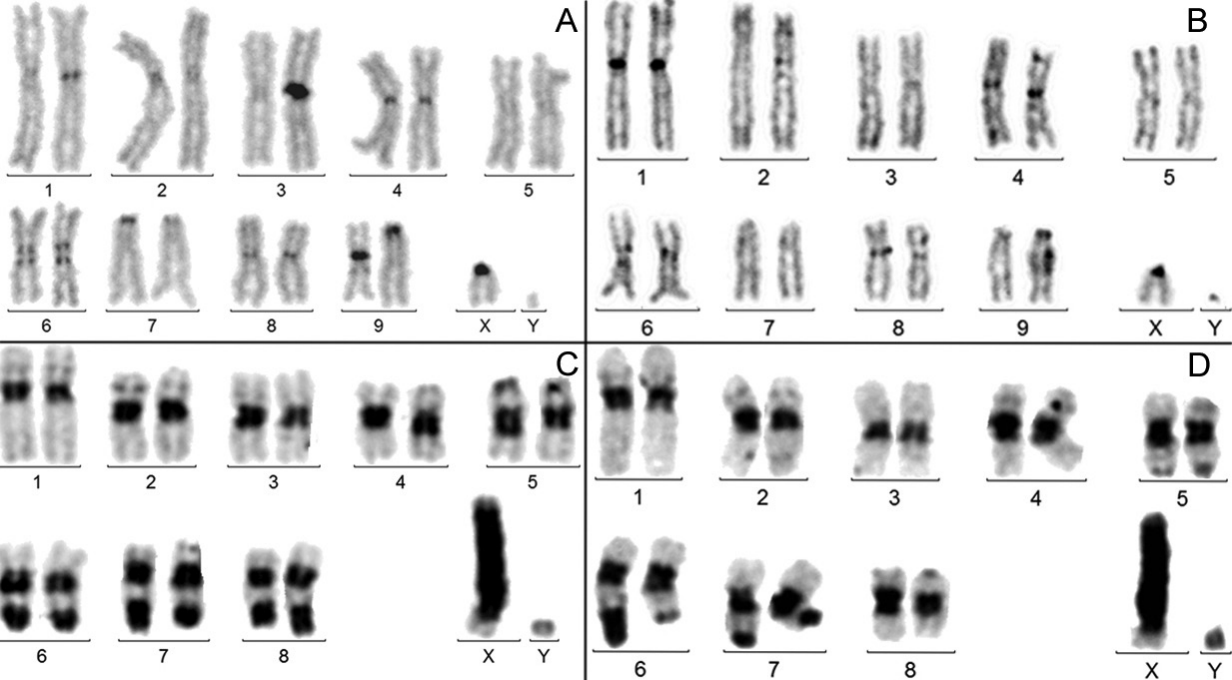
C-banded male karyotypes. **A, B***Melolonthamelolontha***C, D***Macraspistristis*. At contrast with the high variability of autosomes, there is a remarquable stability of the amount of C-banded heterochromatin on the X (average in **A, B** and amplified in **C, D**).

The analysis of most species was generally limited to a few specimens, but short series could be studied for some species. The high variability of both location and amount of heterochromatin is a common place, which was verified here. However, it appeared that variations of heterochromatin are more important on autosomes than on gonosomes. For example, amongst 18 males of *M.melolontha*, the X was always and the Y never C-banded. At contrast, the C-banding of several autosomes was highly polymorphic: it varied in size and could be either present or absent on a single or both homologs (Fig. 5A, B). The same variation of autosomes was observed in 12 females, in which the 2 Xs were always homogenously C-banded. A similar example is provided by the heterochromatin of *M.tristis*, whose heterochromatin is highly and constantly amplified on the X and variable on the autosomes (Fig. 5C, D).

### Heterogeneity of C-banded heterochromatin

**Figure 6.**
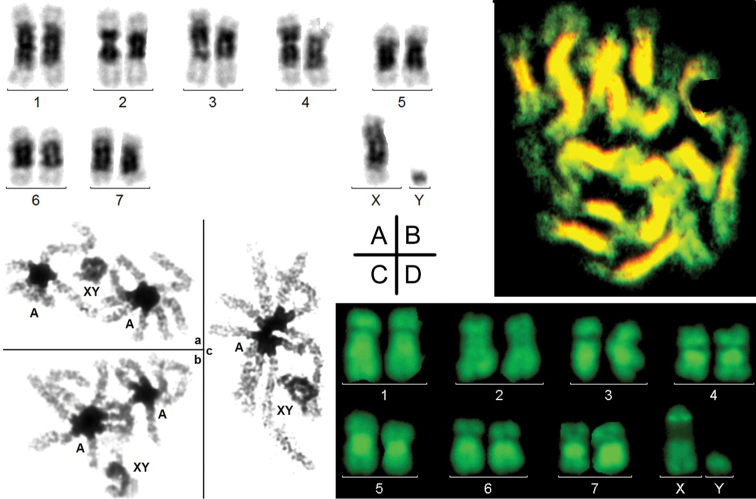
*Criocerisasparagi*. **A** C-banded male karyotype displaying a large heterochromatin amplification in all chromosomes but the Y. **B** Incorporation of BrdU during late S-phase in a female cell: all heterochromatin is homogeneously late replicating (orange staining). The distal fragments of all chromosomes are early replicating (green), which indirectly indicates that there is no Lyonisation of one X. **C** C-banding of 3 spermatocytes (**a, b, c**) at pachynema : autosomal bivalents are at contact and form rosettes after heterochromatin fusion. The sex bivalent is always separated. **D** Q-banded male karyotype: heterochromatin displays at least 3 levels of fluorescence.

The possible heterogeneity of heterochromatin was investigated in the karyotype of *C.asparagi*, in which heterochromatin is strongly amplified on both the X and autosomes. As in most other species, its heterochromatin is homogenously stained after C-banding (Fig. 6A). As usual, compared to mitotic chromosomes, heterochromatin on bivalents at pachynema is much more compacted. Autosomal bivalents frequently form rosettes by fusion of their heterochromatin, while the sex bivalent remains alone (Fig. 6C a, b, c).

**Figure 7.**
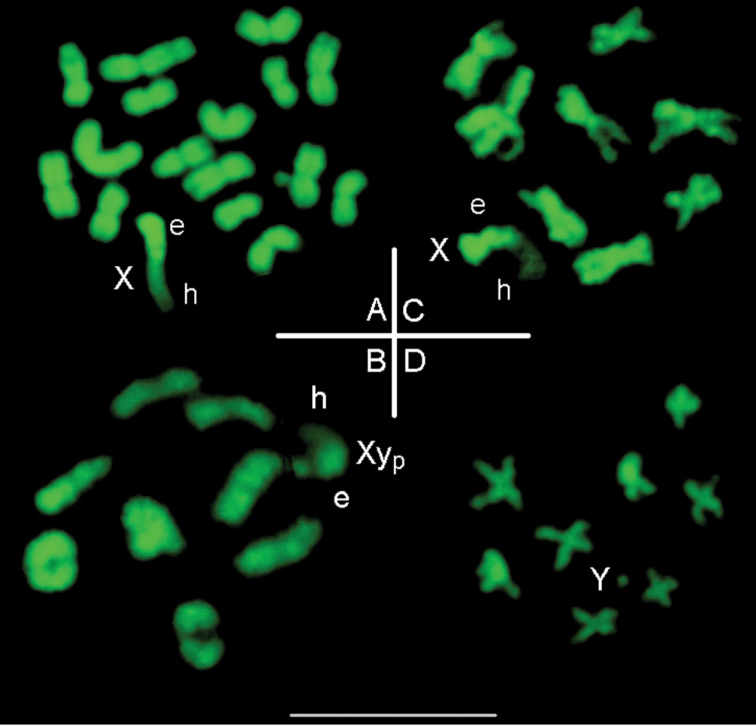
QM-staining of *Macraspistristis* cells. **A** Spermatogonium **B, D** 9,X and 9,Y spermatocytes II **C** 8+Xyp spermatocyte I at diakinesis/metaphase. Heterochromatin, in particular that of the X, displays very different levels of fluorescence. e= euchromatin, h=heterochromatin.

After BrdU incorporation during the late S-phase and acridine orange staining, heterochromatin homogenously fluoresces in orange, indicating its late replication, while early replicating euchromatin fluoresces in green (Fig. 6B). Finally, after staining by quinacrine mustard (Fig. 6D) heterochromatin displays very heterogeneous staining patterns, with at least 3 different levels of fluorescence. Autosomes 3 to 7 share the same fluorescence pattern: dull at centromeres, medium on proximal short arm and brilliant on proximal long arm. The Q-banding of the X is very different with a very large dull and a small brilliant fragment. This relative homogenization of heterochromatin on autosomes, but not on the X is also evidenced in *M.tristis* after quinacrine mustard staining (Fig. 7): heterochromatin is brilliant on autosomes, while a large fragment is dull on the X.

## Discussion

Structural chromosome rearrangements, such as reciprocal and Robertsonian translocations, fissions and intra-changes (inversions, translations, centromere shifts) recurrently occur and differentiate the karyotypes of related species. It seems that in beetles, in which most species possess 20 chromosomes, the karyotype diversification is principally the consequence of intra-changes, but this category of chromosome rearrangements remains difficult to detect, as long as chromosome banding is limited to heterochromatin (Dutrillaux and Dutrillaux 2016). Chromosomal rearrangements create a gametic barrier, and once fixed in a species, they are clonally transmitted to the progeny and can be used for establishing phylogenies. This does not seem to be the case for heterochromatin changes, which are highly frequent within populations and without clear consequence on both reproduction and phenotype. This variation of heterochromatin often affects a variable number of chromosomes, and it is very difficult to decipher both the mechanism inducing these changes and the rules governing their trans-generational transmission. Nevertheless, multiple examples from mammals to insects show that most karyotypes are characterized by a certain heterochromatin pattern, more or less strictly maintained at the level of species, genus or family. This indicates that heterochromatin is not modified and transmitted by each chromosome independently, thus that some regulatory mechanisms exist.

### Hypotheses about the mechanisms of peri-centromeric heterochromatin homogenisation and expansion

The origin of heterochromatin and its highly repeated DNA content, as well as the factors modulating its quantitative and qualitative variations, remain largely unknown, but two main mechanisms have been envisaged.

**1) The recombination process.** As in other animals, peri-centromeric heterochromatin of beetles harbours sequences of repetitive (satellite) DNA (Lorite et al. 2001, 2003, Pons et al. 2002, 2004). Thus, recombination in heterochromatin often consists in exchanges between homologous or pseudo-homologous repeated DNA (Schweizer and Loidl 1987). With time and generations, the repetition of such exchanges would lead to a statistical homogenization of heterochromatin, as regard both its total amount per chromosome and its molecular composition, conferring a characteristic pattern to the whole karyotype. It has been proposed that quantitative variations of heterochromatin could be dependant on external factors, such as altitude, thus would correspond to an adaptation to environmental constraints (Cassagnau 1974). But what kind of exchanges could be in cause? It is well established that meiotic recombination by crossing-over generally avoids heterochromatin and neighbouring regions, which are highly compacted (Fig. 6C). Exchanges (crossing-over) principally occur in euchromatin, which is under-condensed, around the synaptonemal complex (Heyting 1996). Supposing that rare exchanges by crossing-over occur in heterochromatic regions, the presence of repeated DNA would lead to a high probability of asymmetrical exchanges, leading to duplications/deficiencies originating the variation of the amount of heterochromatin between homologous chromosomes. But this would not directly explain the homogenization at the level of the whole karyotype, including the X in particular. For that, exchanges between similar sequences of non-homologous chromosomes would be necessary. In the model of Schweizer and Loidl (1987), which was proposed for telomeric heterochromatin principally, it is supposed that the proximity of telomeres, at early prophase (bouquet stage), might facilitate such pseudo-homologous exchanges. Centromeric heterochromatin is not associated at early prophase, but tight associations recurrently occur later, during the pachytene stage (Dutrillaux et al. 2006 and Fig. 6C). This could also facilitate inter-chromosomal exchanges, but odd numbers of exchanges would lead to form deleterious reciprocal translocations, at difference with exchanges at telomeres. DNA hypo-methylation, particularly of satellite DNA located in heterochromatin, is a strong factor of chromosome instability, leading to breakages and exchanges between both homologous and non-homologous chromosomes (Almeida et al. 1993). Huge variations of DNA methylation, including deep hypo-methylations in heterochromatin, occur at various stages of gametogenesis (Coffigny et al. 1999 and Bernardino-Sgherri et al. 2002). This may favour DNA exchanges and homogenization of heterochromatin at long-term.

**2) *The ocean ridges model.*** This model was proposed to explain the expansion of centromeric repeated DNA (Rudd et al. 2006, Shepelev et al. 2009). It is assumed that centromeric repeated DNA expands by a mechanism recalling the ocean ridges process, with new satellite families appearing in the core centromere and displacing pre-existing satellites towards more distal regions. This process may involve similarly all chromosomes and lead to a fairly homogenous expansion of heterochromatin harbouring satellite DNA in peri-centromere regions of all chromosomes. Mutations could occur later and accumulate, modifying the sequence of the DNA repeats in proportion with their age, i.e., their distance to centromere. For example, a C to T transversion occurring during the expansion of a large DNA repeat would considerably decrease its resistance to denaturation, change the staining properties of the harbouring heterochromatin and even suppress the C-banding.

### Heterochromatin variation in beetles partially supports these hypotheses

Most of the karyotypes of this report share the same tendency for heterochromatin homogenization. The more or less important heterochromatin or C-banding expansion is not totally independent from the systematic classification: for example, most Cerambycidae have small or inconsistent C-bands (Figs 1C, 2B); most Scarabaeidae have average C-bands (Figs 1D, 2A, D, 4A, D), while many Tenebrionidae have very large C-bands (Figs 1B, 3A). However, large heterochromatin amplification may also involve one or a few species only, as *M.hippocastani* in genus *Melolontha* (Fig. 3D), or a genus, as *Crioceris* amongst Criocerinae (personal data) (Fig. 5). According to the above-proposed criterion, amplified heterochromatin was observed in about 25% of species. It was generally similarly amplified on autosomes and the X, which suggests that common mechanisms were at work. However, this expectation, which fits with the result of C-banding only, is obviously over-simple, as shown by our data on *C.asparagi* and *M.tristis*, in which all chromosomes but the Y have amplified heterochromatin in mitotic metaphases. This heterochromatin is homogeneously compacted (shortened) at pachynema, homogeneously C-banded and late replicating, but staining with quinacrine mustard (or DAPI, not shown), known to fluoresce in proportion to the AT richness of DNA, displays huge differences of fluorescence. This demonstrates the presence of different components in different amounts. In these 2 species, the dull fragment (AT-poor) is much larger and the brilliant fragment (AT-rich) much smaller on the X than on the average autosomes. Thus, there is a certain homogenization for the autosomes while the X has a unique fluorescence pattern. A plausible explanation is that heterochromatin amplification depends on an unique mechanism at the cell level, but exchanges occur between autosomes and not or more rarely between gonosomes and autosomes. This interpretation is in agreement with the bivalent behaviour at pachytene stage: all autosomes may form rosettes with tight associations of their heterochromatin, while the sex bivalent remains separated (Fig. 6D), but their eventual exchanges might not be of the same type as crossing-over between euchromatic regions. The small size of the heterochromatin of the Y may depend on an independent erosion mechanism, recalling that proposed for mammals.

In conclusion, there is a large variety of the heterochromatin patterns in the karyotypes of Polyphagan beetles. In spite of inter-individual variations, phylogenetically related taxa tend to share similar characteristics, but exceptions exist: huge amplifications of heterochromatin may affect only a single or all chromosomes of a karyotype and may characterize one or several species in a genus. Thus, heterochromatin constitutes a weak criterion for establishing phylogenetic relationships. A certain homogenisation of the heterochromatin amount and staining capacities exists between the autosomes of a same karyotype. The quantitative, but not qualitative, variations of the heterochromatin of the X grossly follow that of autosomes. At difference, the heterochromatin content of the Y is generally very limited and its variations look largely independent from those of other chromosomes. The concerted variations of autosomes, and the relative independence of the gonosomes, and the Y in particular, may be explained by the strong tendency for fusions of heterochromatin of autosomes, but not gonosomes, at male (and female?) meiotic prophase.
